# Genome wide profiling in oral squamous cell carcinoma identifies a four genetic marker signature of prognostic significance

**DOI:** 10.1371/journal.pone.0174865

**Published:** 2017-04-06

**Authors:** Vui King Vincent-Chong, Iman Salahshourifar, Kar Mun Woo, Arif Anwar, Rozaimi Razali, Ranganath Gudimella, Zainal Ariff Abdul Rahman, Siti Mazlipah Ismail, Thomas George Kallarakkal, Anand Ramanathan, Wan Mahadzir Wan Mustafa, Mannil Thomas Abraham, Keng Kiong Tay, Rosnah Binti Zain

**Affiliations:** 1 Oral Cancer Research and Coordinating Centre, Faculty of Dentistry, University of Malaya, Kuala Lumpur, Malaysia; 2 Department of Oral and Maxillofacial Clinical Sciences, Faculty of Dentistry, University of Malaya, Kuala Lumpur, Malaysia; 3 Department of Biology, Science and Research Branch, Islamic Azad University, Tehran, Iran; 4 Sengenics Sdn Bhd, High Impact Research (HIR) Building, University of Malaya, Kuala Lumpur, Malaysia; 5 Department of Oral and Maxillofacial Surgery, Hospital Kuala Lumpur, Kuala Lumpur, Malaysia; 6 Department of Oral and Maxillofacial Surgery, Hospital Tengku Ampuan Rahimah, Klang, Selangor Darul Ehsan, Malaysia; 7 Department of Oral Surgery, Hospital Umum Kuching, Kuching, Sarawak, Malaysia; Georgetown University, UNITED STATES

## Abstract

**Background:**

Cancers of the oral cavity are primarily oral squamous cell carcinomas (OSCCs). Many of the OSCCs present at late stages with an exceptionally poor prognosis. A probable limitation in management of patients with OSCC lies in the insufficient knowledge pertaining to the linkage between copy number alterations in OSCC and oral tumourigenesis thereby resulting in an inability to deliver targeted therapy.

**Objectives:**

The current study aimed to identify copy number alterations (CNAs) in OSCC using array comparative genomic hybridization (array CGH) and to correlate the CNAs with clinico-pathologic parameters and clinical outcomes.

**Materials and methods:**

Using array CGH, genome-wide profiling was performed on 75 OSCCs. Selected genes that were harboured in the frequently amplified and deleted regions were validated using quantitative polymerase chain reaction (qPCR). Thereafter, pathway and network functional analysis were carried out using Ingenuity Pathway Analysis (IPA) software.

**Results:**

Multiple chromosomal regions including 3q, 5p, 7p, 8q, 9p, 10p, 11q were frequently amplified, while 3p and 8p chromosomal regions were frequently deleted. These findings were in confirmation with our previous study using ultra-dense array CGH. In addition, amplification of 8q, 11q, 7p and 9p and deletion of 8p chromosomal regions showed a significant correlation with clinico-pathologic parameters such as the size of the tumour, metastatic lymph nodes and pathological staging. Co-amplification of 7p, 8q, 9p and 11q regions that harbored amplified genes namely CCND1, EGFR, TPM2 and LRP12 respectively, when combined, continues to be an independent prognostic factor in OSCC.

**Conclusion:**

Amplification of 3q, 5p, 7p, 8q, 9p, 10p, 11q and deletion of 3p and 8p chromosomal regions were recurrent among OSCC patients. Co-alteration of 7p, 8q, 9p and 11q was found to be associated with clinico-pathologic parameters and poor survival. These regions contain genes that play critical roles in tumourigenesis pathways.

## Introduction

Globally, oral and oropharyngeal cancer is ranked as the sixth most common cancer with an estimated 300,000 new cases being reported in 2012 [1]. Although globally oral cancer is a male-predominant disease [2], incidence of oral cancer in Malaysia varies according to gender and ethnicity [3]. According to the National Cancer Registry Statistics in Malaysia, there was a higher incidence of oral cancer reported in Indian and Malay females. In contrast, for the Chinese population, there was a high oral cancer incidence in males. The higher prevalence of oral cancer in Indian female population may be related to the predominant lifestyle habits such as betel quid chewing [4] among this group. Despite the advances in diagnosis and therapeutic approaches, the mortality and morbidity rates have not improved over the past decades [5]. Copy number alterations promote genetic instability in cancer and lack of improvement in the clinical outcomes most probably reflects the paucity in the knowledge that explains how genetic instabilities in oral cancer contribute in oral carcinogenesis [6, 7]. Moreover, molecular heterogeneity is another issue that should be kept in mind [8].

Oral carcinogenesis is a complex process, resulting from a multistep pathway with accumulation of genetic alterations [7]. Copy number alterations (CNAs) that include amplifications and deletions result in activation of proto-oncogenes and inactivation of tumour suppressor genes, respectively [9]. Several recurrent CNAs have been reported in OSCC by many authors [10–13], but how these CNAs play a role in the pathogenesis of OSCC has not been thus far elucidated. Profiling of CNAs using high-throughput methods provides advanced tools to discover potential biomarkers that could be used for predictive, prognostic and diagnostic approaches [14, 15].

The pattern of CNAs as biomarkers have remarkable significance due to their great impact related with diseases outcome and personalized medicine [15]. Therefore, the identification of the effective biomarkers for prognosis and diagnosis is an early step in the plan for molecular sub-classification that underlies the pathophysiology of the disease. These specific molecular classifications may have the potential to predict early disease and in deciding the patient’s treatment including personalized medicine (targeted gene therapy) [15]. Cervical lymph node metastasis (LNM) is a predictor of poor prognosis in OSCC [16, 17]. The ability to accurately predict lymph node metastases in OSCC patients will enable the clinician to plan the appropriate treatment. However, the CNAs in OSCC and its association with clinico-pathological parameters and clinical outcome remain undetermined. Hence, we aimed to identify recurrent CNAs and their clinical and prognostic impacts in OSCC using high-resolution array CGH. High-resolution array CGH could aid in the identification of candidate genes/regions that may drive the development of oral cancer.

## Materials and methods

In this study, tumour samples selected were SCCs derived from within the oral cavity consisting of the anterior two-thirds of the tongue, the buccal mucosa, alveolar ridge, lip, floor of the mouth and hard palate (C00, 02–06) and excluded the base of tongue (C01) and other head and neck sites such as oropharynx, hypopharynx and larynx due to their varied etiologic, genetic, clinical characteristics and prognosis [18, 19]. For example, the principal etiological factors for oral cancer are tobacco smoking, alcohol drinking and betel quid chewing whereas majority of the oropharyngeal cancers are HPV-related. Apart from that, Chung et al. [20] observed that OSCC is more heterogeneous in terms of their genetic and molecular expression as compared to squamous cell carcinomas derived from other head and neck regions (oropharynx, hypopharynx, and larynx). In view of this, we have grouped OSCC of all these sub-sites (C00, C02-06) as a single oral cancer site in this current study.

### Tumour samples

A total of 75 OSCC fresh-frozen tissue samples were included for the genome wide array CGH analysis. Sixty-six overlapping OSCC samples were used for validation of the CNAs that resulted from array CGH data using quantitative real-time PCR analysis. Fresh-frozen OSCC tissue samples and the related socio-demographic (risk habits, gender, age group) and clinico-pathologic data (site of lesion, tumour size, lymph node status and tumour staging) were acquired from the Malaysian Oral Cancer Database and Tissues Bank System (MOCDTBS) coordinated by the Oral Cancer Research and Coordinating Centre, University of Malaya [21]. All the OSCC samples recruited in this study had been tested for infection with Human papillomavirus (HPV) 16 and 18 using the HPV GenoArray (Hybribio Ltd, Hong Kong) and all were found to be negative for both types (unpublished data). The socio-demographic and clinico-pathologic parameters of the OSCC samples are listed in Table 1. The International Classification of Disease (ICD-10), developed by World Health Organization (WHO) was used to categorize the OSCC samples according to the anatomical subsites. Tumour staging was done according to the criteria by The American Joint Committee on cancer staging [22]. All the OSCC samples that were included in this study were histologically confirmed by oral pathologists. Approval for this study was granted by the Medical Ethics Committee (MEC), Faculty of Dentistry, University of Malaya vide MEC code no: DF0306/ 001/(L) and DF OS1007/0048(P). All the methodology employed in this study was in accordance with International Conference on Harmonisation–Good Clinical Practice (ICH-GCP) guideline for good clinical practice and the Declaration of Helsinki.

**Table 1:** Socio-demographic and clinico-pathologic parameters of the 75 OSCC cases involved in array CGH study.

Variables	Category	No. of patients (%)
Total		75
Gender	Male	26 (34.7)
	Female	49 (65.3)
Age (years)	< 45	12 (16.0)
	≥ 45	63 (84.0)
Smoking	No	52 (69.3)
	Yes	23 (30.7)
Drinking	No	64 (85.3)
	Yes	11 (14.7)
Betel quid chewing	No	40 (53.3)
	Yes	35 (46.7)
Tumour site	Tongue	24 (32.0)
	Non-tongue*	51 (68.0)
Tumour size	T1-T2	45 (60.0)
	T3-T4	30 (40.0)
Lymph node metastasis	Negative	38 (50.7)
	Positive	37 (49.3)
pTNM Staging	Early stage	26 (34.7)
	Advanced stage	49 (65.3)
Overall survival	Range	1–114 months
	Median	21.0 months
	Mean	26.24 months

* Non-tongue = Buccal mucosa, gingiva, lip, floor of mouth and plate

### Histopathology and array CGH analysis

Histological assessment was carried out on haematoxylin and eosin (H&E) stained frozen tissue specimens mounted in optimal cutting temperature (OCT) compound. The sections were analysed to determine percentage of tumour content. Tissues that did not contain 70% tumour were macro-dissected to gain areas with ≥ 70% of tumour content for DNA extraction. Extraction of DNA was carried out using DNeasy Blood & Tissue Kit (Qiagen, Hilden, Germany) according to the manufacturer’s instructions.

A customized array CGH platform was designed for OSCC based on our previous research [11, 23] and previously reported candidate regions/genes for OSCC. This customized oligonucleotide array CGH (8x60k) was manufactured by Agilent Technologies, CA, USA. Thereafter, genome-wide profiling was completed based on the manufacturer's instructions (version 5.0, June 2007) by Oxford Gene Technology, Oxford, UK. Genomic DNA of tumour samples was fragmented by enzymatic digestion. Subsequent steps included sample labelling, probe purification, microarray hybridization, washing and scanning. For each array CGH profiling, 1.5 μg of DNA (gDNA) from each of the test samples and commercially obtained gender matched pooled blood gDNA sample (Promega Corporation, WI, USA) were obtained and labelled with fluorescence Cy5 and Cy3 dyes in dye-swap protocol using the CytoSure Genomic DNA labelling kit (Oxford Gene Technology, Oxford, UK), respectively. Probe purification was done using Microcon YM-30 filters (Merck Millipore, MA, USA). This was followed by probe denaturation and pre-annealing with Cot-1 DNA. Constant rotation at 20 rpm (65°C for 40 hours) completed the hybridization process. Slides were washed after the hybridization process conforming to the manufacturer’s instructions and scanned using a DNA Microarray Scanner (Agilent Technologies, CA, USA). Feature Extraction software, version 10.7.3.1 (Agilent Technologies, CA, USA) was used to generate the signal intensities in the text file per array. The data from the text file was segmented using a modified Circular Binary Segmentation (CBS) algorithm [24]. The CNAs were recognized using CytoSure Interpret software version 4.2.5 (Oxford Gene Technology, Oxford, UK) based on the application of log2 intensity ratios of sample to reference (Cy3/Cy5: log2-ratios above 0.3 for amplifications and below -0.6 for deletions). CNAs genomic positions (start and end) along with list of cytobands were annotated based on the human genome assembly version GRCh37/hg19. The microarray data have been deposited in the Gene Expression Omnibus (GEO) database with the accession number of GSE89924. CNAs were classified irrespective of the sizes which may have gene-rich regions with possibility of being pathogenic. CNAs with closest overlap regions and their redundancies with in each cytoband were considered to calculate frequency of amplification and/or deletions. Overall percentages were calculated by dividing the frequency of amplification and/or deletion in each cytoband by total number of CNAs identified. Cytobands were ranked according to the percentage, a cutoff of 8% was applied to highlight significant CNAs and also focus on highly altered genes in CNA. Significant CNAs identified by above cutoff were compared to data from The Cancer Genome Atlas (TCGA) [25] and International Cancer Genome Consortium (ICGC) [26].

### Pathway and network analysis

The annotated genes within the copy number altered regions that had a frequency of 8% were subjected to gene pathway/network and biological function (diseases, molecular and cellular functions) analysis using Ingenuity Pathway Analysis (IPA) software (Ingenuity Systems, CA, USA). The default setting from the software was used to map the CNA associated genes to the reference set of direct and indirect relationships. Next, relevant input to the gene list such as the molecular networks and biological functions were generated by the software algorithm. The significance of the gene annotation with a p-value less than 0.05 was determined with right-tailed Fisher’s exact test.

### Copy number analysis by the TaqMan PCR assay

Copy number analysis was done on 66 OSCCs using TaqMan Copy Number Assay: LRP12 (Hs01987319_cn), FSCN1 (Hs03631914_cn), EGFR (Hs02309320_cn), CCND1 (Hs02226007_cn), CHL1 (Hs02163529_cn), TPM2 (Hs01060645_cn), CLPTM1L (Hs01133209_cn), CSMD1 (Hs03683117_cn) (Applied Biosystems, Foster City, CA, USA). The commercially male and female-pooled blood gDNA samples (Promega Corporation, WI, USA) served as calibrator controls. PCR was done in a total volume of 20 μl consisting of 4 μl of genomic DNA(5 ng/μl), 10 μl of 2× TaqMan^®^ Genotyping Master Mix (Applied Biosystems, CA, USA), 1 μl of 20X TaqMan Copy number assay, 1 μl of 20X TaqMan copy number reference assay (RNAse P) and 4 μl of nuclease free water. Quantitative PCR was performed on an ABI 7500 Fast Real Time PCR System (Applied Biosystems, CA, USA) using the manufacturer’s PCR conditions as follows: initial denaturation at 95°C for 10 minutes followed by 40 cycles of denaturation for 15 seconds at 95°C and annealing for 60 seconds at 60°C.

The values of copy number for each sample were normalized using RNAase P as a reference control with 2 copies in the human genome. Copy number was quantified using the equation 2 x (2−ΔΔCt), comparative CT (ΔΔCT) relative quantitation method [27]. Target and reference assays that were used for copy number calculation were derived from the mean of triplicate, RNase P and the calibrator samples. The calculated relative quantity was multiplied by a base copy number of 2 to obtain the copy number value. The copy number of selected CNA associated genes were then classified into three groups, deletion (< 1.0), amplification (> 2.0 copies) and no change (> 1.0 and ≤ 2.0 copies) [28, 29].

### Selection of the cut-off point for LRP12, FSCN1, EGFR, CCND1, CHL1, TPM2, CLPTM1L, CSMD1 genes

The clinico-pathologic data was first dichotomized based on the survival status of the OSCC patients (alive vs dead). Receiver operating characteristic (ROC) curve analysis was used to determine the best cut-off score for LRP12, FSCN1, EGFR, CCND1, CHL1, TPM2, CLPTM1L and CSMD1 genes copy number to survival status using 0, 1 criterion [30]. For copy number alterations (scores) of the LRP12, FSCN1, EGFR, CCND1, CHL1, TPM2, CLPTM1L and CSMD1 genes, the sensitivity and specificity of each score was plotted to generate various area under the ROC curves (AUC) against survival status (alive vs dead). The score that was closest to the point with maximum sensitivity and specificity was selected as the cut-off value. The copy number alteration scores were divided into amplifications/deletions and no change where no change was the score below or equal to the cut-off value, while amplifications/deletions were the scores above the cut-off value.

### Statistical analysis

The chi-square (or Fisher exact where appropriate) statistic was used to test the associations between the selected CNAs (amplification of chromosome 3q, 8q, 7p, 9p, 11q and deletion of 3p and 8p) and clinico-pathologic parameters. The Mann-Whitney U test was used to compare copy number changes of the candidate genes (LRP12, FSCN1, EGFR, CCND1, CHL1, TPM2, CLPTM1L, CSMD1) between OSCC and non-cancer tissues. The Kaplan-Meier analysis was used to ascertain the prognostic significance of these CNAs and candidate genes of the chromosomes studied. In order to further test whether any of the selected CNAs and the associated genes which showed significant association from the Kaplan-Meier analysis, the Multivariate Cox Regression analysis was further employed. All statistical analyses were performed using the SPSS statistical package (SPSS version 12.0, IL, USA) and the p-values < 0.05 was considered significant.

## Results

### Copy number alterations

**Fig 1:**
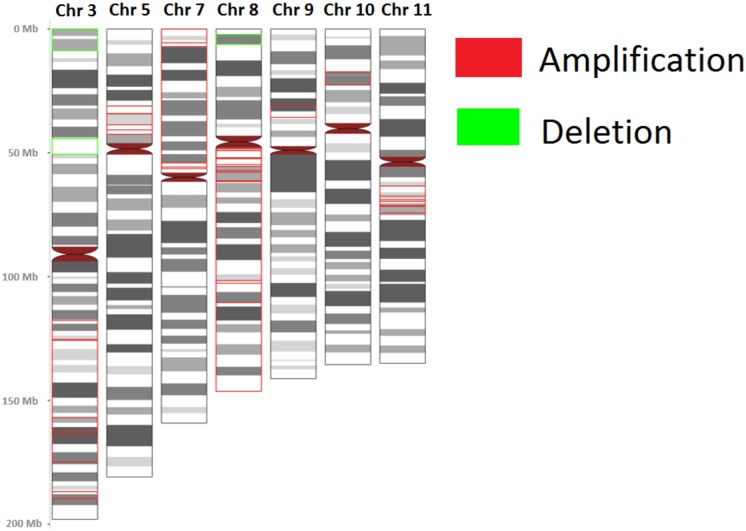
The ideogram of amplifications and deletions identified in this study using array CGH.

**Fig 2:**
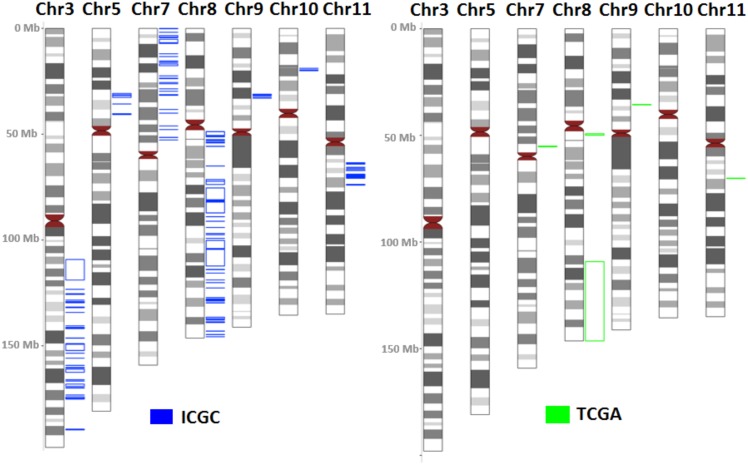
The ideogram of CNAs identified representing intersection of cytoband CNAs from TCGA and ICGC studies.

The regions with a frequency of copy number alterations that was ≥ 8% were reported in this study. In array CGH analysis, 26 amplified and 3 deleted chromosomal regions were found (Table 2 and Fig 1). The number of occurrences, size of the start genome position and end genome position of the CNAs are illustrated in Table 2. In the whole genome wide profiling dataset, amplifications outnumbered deletions. Amplifications in 3q, 5p, 7p, 8q, 9p, 10p, 11q and deletions in 3p and 8p chromosomal regions were recurrent. Amplification in 8q22.3-q23.1 and deletion in 3p21.31 were the most common findings, accounting for 18.7% and 9.3% of all samples, respectively (Table 2). Chromosomal regions 3q, 8q and 11q depicted the largest number of CNAs (Table 2 and Fig 1). There were 11 and 21 CNAs identified from the current study that shared similarities with the TGCA of the oral cancer array CGH OSCC study and the International Cancer Genome Consortium (ICGC) respectively (Fig 2 and S1 Table).

**Table 2:** Amplified and deleted regions detected in 75 OSCC samples.

Cytoband	Median start position	Median end position	CNAs (AMP/DEL)	Number of sample	Percentage % (n = 75)
8q22.3-q23.1	102681371	109392898	AMP	14	18.7
7p11.2	54033427	56399307	AMP	12	16
8q12.1	57356305	61290015	AMP	11	14.7
5p13.2-p13.1	34263518	42496863	AMP	11	14.7
9p21.1-p13.3	30940311	35689407	AMP	11	14.7
8q21.13-q21.2	48435432	57172822	AMP	10	13.3
7p22.1-p11.2	7091279	55728080	AMP	9	12
8q11.21-q12.1	48435432	102647978	AMP	9	12
5p13.3-p13.2	31085740	34171734	AMP	9	12
11q13.1-q13.2	63411714	67465752	AMP	9	12
8q23.1-q24.11	48435432	51961330.5	AMP	9	12
8q24.13	48435432	146301585	AMP	8	10.7
8q24.3	49084980	54788562	AMP	8	10.7
3q13.32-q21.2	117659990	125404921	AMP	7	9.3
8q21.2-q21.3	49084980	52283007	AMP	7	9.3
7p22.3-p22.1	16324	5554669	AMP	7	9.3
5p13.1	38651455	40760663	AMP	7	9.3
11q13.3-q13.4	69592775	71296836	AMP	7	9.3
3q25.31-q26.1	156865802	162501514	AMP	7	9.3
3q27.3-q28	186822642	189711307	AMP	7	9.3
8q24.12-q24.13	57804398	61290015	AMP	6	8
11q13.3	68889918	69589223.5	AMP	6	8
11q13.4	71627053	74357770	AMP	6	8
10p13-p12.2	17275747.5	22617571	AMP	6	8
3q21.2-q26.1	125683802	162501514	AMP	6	8
3q26.1-q26.31	164035254	174942968.5	AMP	6	8
3p21.31	47076499	49558487	DEL	7	9.3
3p26.3-p26.1	64052	5256910	DEL	7	9.3
8p23.2	3680600	3841195	DEL	6	8

### Association of the copy number alterations with clinico-pathologic parameters

Amplification of the chromosome 7p was significantly associated with both the tumour size (T1-T2: 31.1% vs T3-T4: 70%, p = 0.001) and staging (early stages: 26.9% vs advanced stages: 57.1%, p = 0.013). Amplification in the long arm of chromosome 8 (early stage: 15.4% vs advanced stages: 51.1%, p = 0.003), and 11 (early stages: 7.7% vs advanced stages: 28.6%, p = 0.036) was associated with staging. In addition, deletion in the short arm of the chromosome 8 was found to be significantly associated with pathologic staging, (early stages: 11.5% vs advanced stages: 38.8%, p = 0.014). Amplification in the short arm of chromosome 9 was significantly associated with lymph node metastasis (LNM negative: 5.3% vs LNM positive: 24.3%, p = 0.02) (Table 3). The association of amplification of chromosomes 7p, 8q, 9p and 11q with the size of the tumour, metastatic lymph nodes and pathological staging prompted us to combine these CNAs as a genetic signature to increase clinical significance in OSCC patients. Co-amplification of ≥ 1 of these CNAs within the genetic signature were found to be associated with tumour sizes (T1-T2: 55.6% vs T3-T4: 83.3%, p = 0.012), lymph node metastasis (LNM negative: 52.6% vs LNM positive: 81.1%, p = 0.009) and pathologic staging (early stages: 42.3% vs advanced stages: 79.6%, p = 0.001) (Table 3).

**Table 3:** Association of chromosomes 7p, 8q, 9p, 11q, 8p and the combination of chromosomes 7p, 8q, 9p, 11q with clinico-pathologic parameters in OSCC.

Variables	Category	No. of patients (%)	7p			8q			11q			9p			(chr 7p+8q+9p+11q)			8p		
			No change	Gain	P value	No change	Gain	P value	No change	Gain	P value	No change	Gain	P value	No marker	≥1 marker	P value	No change	Loss	P value
**Total**		75	40	35		46	29		59	16		64	11		25	50		26	49	
**Gender**	**Male**	26 (34.7)	17 (65.4)	9 (34.6)	0.128	14 (53.8)	12 (46.2)	0.332	18 (69.2)	8 (30.8)	0.146	22 (84.6)	4 (15.4)	1.000	9 (34.6)	17 (65.4)	0.864	15 (57.7)	11 (42.3)	0.072
	**Female**	49 (65.3)	23 (46.9)	26 (53.1)		32 (65.3)	17 (34.7)		41 (83.7)	8 (16.3)		42 (85.7)	7 (14.3)		16 (32.7)	33 (67.3)		38 (77.6)	11 (22.4)	
**Age (years)**	**< 45**	12 (16.0)	5 (41.7)	7 (58.3)	0.377	6 (50.0)	6 (50.0)	0.519	8 (66.7)	4 (33.3)	0.271	9 (75.0)	3 (25.0)	0.368	3 (25.0)	9 (75.0)	0.74	8 (66.7)	4(33.3)	0.739
	**≥ 45**	63 (84.0)	35 (55.6)	28 (44.4)		40 (63.5)	23 (36.5)		51 (81.0)	12 (19.0)		55 (87.3)	8 (12.7)		22 (34.9)	41 (65.1)		45 (71.4)	18 (28.6)	
**Smoking**	**No**	52 (69.3)	27 (51.9)	25 (48.1)	0.713	33 (63.5)	19 (36.5)	0.569	41 (78.8)	11 (21.2)	1.000	41 (78.8)	11 (21.2)	0.015*	17 (32.7)	35 (67.3)	0.859	38 (73.1)	14 (26.9)	0.491
	**Yes**	23 (30.7)	13 (56.5)	10 (43.5)		13 (56.5)	10 (43.5)		18 (78.3)	5 (21.7)		23 (100.0)	0 (0.0)		8 (34.8)	15 (65.2)		15 (65.2)	8 (34.8)	
**Drinking**	**No**	64 (85.3)	35 (54.7)	29 (45.3)	0.571	37 (57.8)	27 (42.2)	0.186	51 (79.7)	13 (20.3)	0.692	53 (82.8)	11 (17.2)	0.351	20 (31.3)	44 (68.8)	0.49	45 (70.3)	19 (29.7)	1.000
	**Yes**	11 (14.7)	5 (45.5)	6 (54.5)		9 (81.8)	2 (18.2)		8 (72.7)	3 (27.3)		11 (100.0)	0 (0.0)		5 (45.5)	6 (54.5)		8 (72.7)	3 (27.3)	
**Betel quid chewing**	**No**	40 (53.3)	21 (52.5)	19 (47.5)	0.877	23 (57.5)	17 (42.5)	0.466	31 (77.5)	9 (22.5)	0.792	34 (85.0)	6 (15.0)	0.93	11 (27.5)	29 (72.5)	0.252	27 (67.5)	13 (32.5)	0.520
	**Yes**	35 (46.7)	19 (54.3)	16 (45.7)		23 (65.7)	12 (34.3)		28 (80.0)	7 (20.0)		30 (85.7)	5 (14.3)		14 (40.0)	21 (60.0)		26 (74.3)	9 (25.7)	
**Tumour site**	**Tongue**	24 (32.0)	14 (58.3)	10 41.7)	0.552	12 (50.0)	12 (50.0)	0.167	20 (83.3)	4 (16.7)	0.499	22 (91.7)	2 (8.3)	0.486	6 (25.0)	18(75.0)	0.294	18 (75.0)	6 (25.0)	0.572
	Non-tongue**	51 (68.0)	26 (51.0)	25 (49.0)		34 (66.7)	17 (33.3)		39 (76.5)	12 (23.5)		42 (82.4)	9 (17.6)		19 (37.3)	32 (62.7)		35 (68.6)	16 (31.4)	
**Tumour size**	**T1-T2**	45 (60.0)	31 (68.9)	14 (31.1)	0.001*	32 (71.1)	13 (28.9)	0.033*	38 (84.4)	7 (15.6)	0.135	40 (88.9)	5 (11.1)	0.330	20 (44.4)	25 (55.6)	0.012*	35 (77.8)	10 (22.2)	0.098
	**T3-T4**	30 (40.0)	9 (30.0)	21 (70.0)		14 (46.7)	16 (53.3)		21 (70.0)	9 (30.0)		24 (80.0)	6 (20.0)		5(16.7)	25 (83.3)		18 (60.0)	12 (40.0)	
**Lymph node metastasis**	**Negative**	38 (50.7)	24 (63.2)	14 (36.8)	0.084	27 (71.1)	11 (28.9)	0.080	33 (86.8)	5 (13.2)	0.080	36 (94.7)	2 (5.3)	0.020*	18 (47.4)	20 (52.6)	0.009*	30 (78.9)	8 (21.1)	0.110
	**Positive**	37 (49.3)	16 (43.2)	21 (56.8)		19 (51.4)	18 (48.6)		26 (70.3)	11 (29.7)		28 (75.7)	9 (24.3)		7 (18.9)	30 (81.1)		23 (62.2)	14 (37.8)	
**pTNM Staging**	**Early stage**	26 (34.7)	19 (73.1)	7 (26.9)	0.013*	22 (84.6)	4 (15.4)	0.003*	24 (92.3)	2 (7.7)	0.036*	25 (96.2)	1 (3.8)	0.085	15 (57.7)	11 (42.3)	0.001*	23 (88.5)	3 (11.5)	0.014*
	**Advanced stage**	49 (65.3)	21 (42.9)	58 (57.1)		24 (49.0)	25 (51.1)		35 (71.4)	14 (28.6)		39 (79.6)	10 (20.4)		10 (20.4)	39 (79.6)		30 (61.2)	19 (38.8)	

*Significant p—value were highlighted in bold

**Non-tongue = Buccal mucosa, gingiva, lip, floor of mouth and plate

### Association of the copy number alterations with clinical outcomes

**Fig 3:**
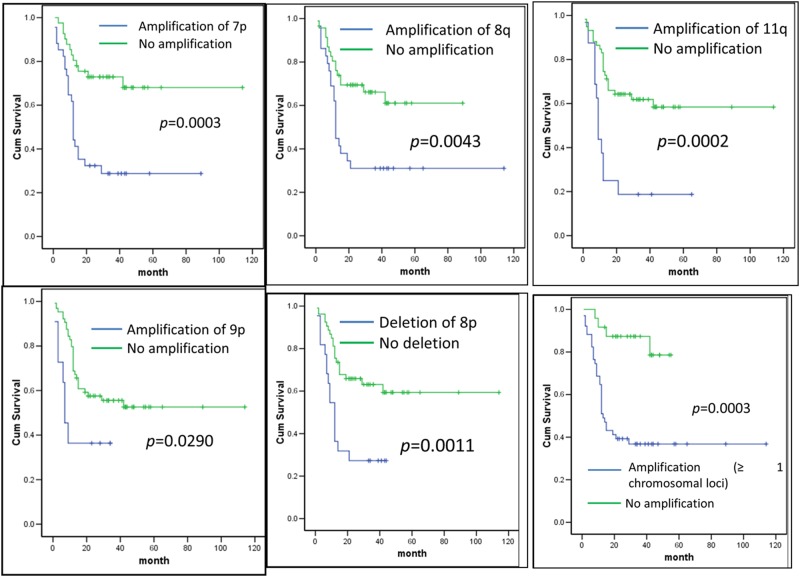
Overall survival curves were analyzed according to amplification of chromosome 7p, 8q, 9p, 11q and deletion of chromosome 8p and ≥ 1 of chromosome 7p, 8q, 9p and 11q using Kaplan-Meier estimate with log-rank test.

Three-year survival rates for amplification and non-amplification of all chromosomes are summarized in S2 Table. Using Kaplan Meier analysis (Fig 3), amplification of chromosomes 7p, 8q, 9p, 11q and deletion of 8p was significantly associated with poor prognosis. However, after using multivariate analysis and controlling for other confounders (after adjustment for selected socio-demographic and clinico-pathologic data), all associations obtained in the Kaplan Meier analysis were not significant except for chromosome 11 (S3 Table). Thus, only chromosome 11q can be accepted as an independent prognostic marker based on the Multivariate analysis (S3 Table).

When amplification of any 1 or more (≥ 1) of the chromosomes 7p, 8q, 9p, 11q was considered, both Kaplan-Meier (Fig 3) and Multivariate Cox regression analysis (S3 Table) revealed that co-amplification of ≥ 1 of these CNAs within the genetic signature was found to be associated with poor prognosis (HRR = 3.554, 95% C1 1.161–10.886, p = 0.026) after adjustment for selected socio-demographic and clinico-pathologic data of OSCC (S2 Table). This result showed that in addition to amplification of chromosome 11, co-amplification of ≥ 1 of these CNAs within the genetic signature is also an independent prognostic marker.

### TaqMan copy number assay of LRP12, TPM2, EGFR, FSCN1, CCND1, CLPTM1L, CHL1 and CSMD1

**Fig 4:**
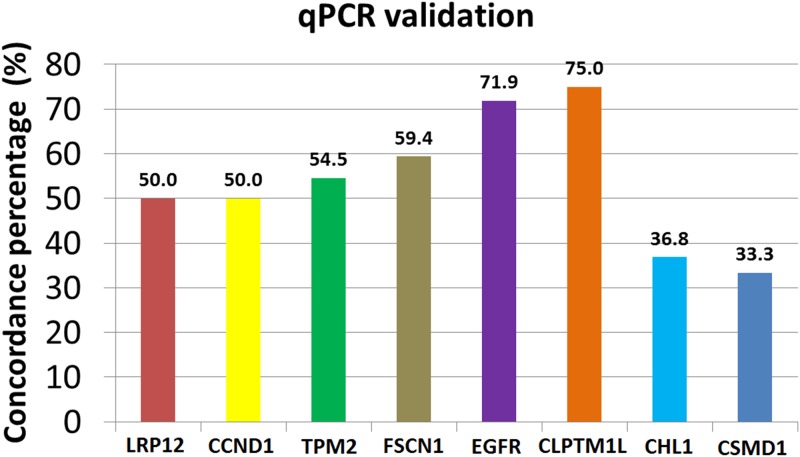
Concordance percentage for amplification of LRP12 (chr 8q), CCND1 (chr 11q), TPM2 (chr 9p), FSCN1 (chr 7p), EGFR (chr 7p), CLPTM1L (chr 5p) and deletion of CHL1 (chr 3p) and CSMD1 (chr 8p) identified using array CGH and validated using qPCR copy number analysis in OSCC samples.

Several candidate genes within the chromosomal regions that showed changes in the copy number were validated using qPCR analysis (Fig 4). Out of 26 samples that showed amplification of chromosome 8q22.3 (LRP12) in array CGH, 13 (50%) samples showed amplification in the qPCR copy number assay validation. Approximately 50% (7/14), 54.5% (6/11), 59.4%, 71.9% and 75% of the samples that showed amplification in array CGH analysis for CCND1 (chromosome 11q13.3), TPM2 (chromosome 9p13.3), FSCN, EGFR (chromosome 7p11.2) and CLPTM1L (chromosome 5p15.33) respectively were validated in qPCR copy number analysis. As for the deletion CNAs, approximately 33.3% (7/21) and 36.8% (7/19) of the samples that showed deletions in array CGH analysis for CSMD1 (chromosome 8p23.2) and CHL1 (chromosome 3p26.3) respectively were validated in qPCR copy number analysis (Fig 4).

**Fig 5:**
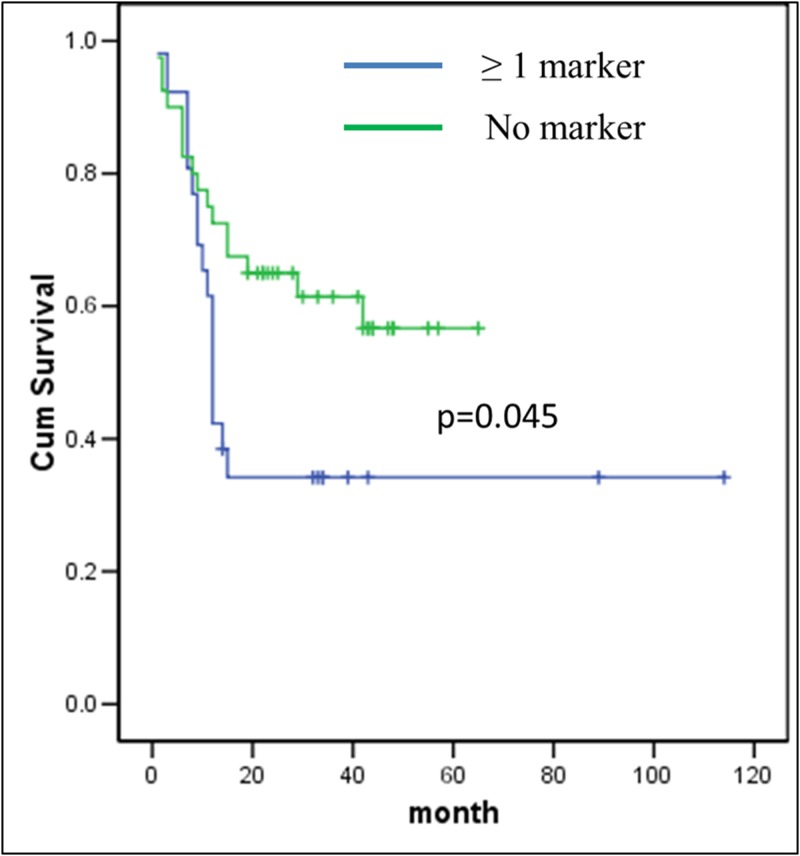
Overall survival curves were analyzed according to ≥ 1 of genetic marker (EGFR, CCND1, TPM2 and LRP12) using Kaplan-Meier estimate with log-rank test.

The chi-square analysis results showed that LRP12, TPM2, EGFR, FSCN1, CCND1, CLPTM1L, CHL1 and CSMD1 genes were not associated with socio-demographic and clinico-pathologic parameters. Additionally, Kaplan-Meier survival analysis did not show statistical significance between LRP12, TPM2, FSCN1, CCND1, CLPTM1L, CHL1 and CSMD1 genes and poor prognosis. Only amplification of the EGFR showed a trend towards association with poor prognosis (p = 0.060). Moreover, the combination of four candidate genes namely EGFR, LRP12, TPM2 and CCND1 located on 7p, 8q, 9p and 11q respectively were subjected to the statistical analysis. The combined AUC for all the markers was 0.621 which is higher than the single genetic markers. The OSCC patients were divided into two groups (Group 1 & 2). Group 1 included those patients who had a cumulative score of 0 markers while those with a cumulative score of 1, 2, 3, or 4 markers were placed under group 2. A significant difference between groups 1 and 2 (p = 0.045) (Fig 5) was observed in the Kaplan-Meier survival curves. It was also observed that the 4 combined genetic markers remained as an independent prognostic factor with a hazard risk ratio (HRR = 2.34) towards death in patients with amplification of 1 or more markers after adjustment for socio-demographic and clinico-pathologic parameters (Table 4) using Cox regression multivariate model.

**Table 4:** Multivariate cox regression model analysis of four combined genetic markers consisting of EGFR, TPM2, CCND1 and LRP12 in OSCC overall survival.

Variables	Category	No. of patients (%)	Multivariate Logistic regression**		
Total		66	HRR	95% CI	p value
Four combined genetic markers	no marker	40 (60.6)	1.00†		**0.038**
	≥ 1 marker	26 (39.4)	2.343	1.047–5.244	
Gender	Male	24 (36.4)	1.00†		0.508
	Female	42 (63.6)	1.4	0.517–3.794	
Age (years)	< 45	12 (18.2)	1.00†		0.977
	≥ 45	54 (81.8)	1.016	0.331–3.119	
Smoking	No	45 (68.2)	1.00†		0.589
	Yes	21 (31.8)	0.744	0.254–2.178	
Drinking	No	57 (86.4)	1.00†		0.398
	Yes	9 (13.6)	1.899	0.429–8.406	
Betel quid chewing	No	35 (53.0)	1.00†		0.107
	Yes	31 (47.0)	2.089	0.852–5.122	
Tumour size	T1-T2	37 (56.1)	1.00†		**0.018**
	T3-T4	29 (43.9)	3.36	1.23–9.177	
Lymph node metastasis	Negative	33 (50.0)	1.00†		0.228
	Positive	33 (50.0)	1.843	0.682–4.983	
pTNM Staging	Early	22 (33.3)	1.00†		0.322
	Advanced	44 (66.7)	2.27	0.449–11.484	
Differentiation	Well	27 (40.9)	1.00†		0.299
	Moderate and poor	39 (59.1)	1.526	0.687–3.388	

CI: confidence interval

^†^ Reference category

Significant p—value were highlighted in bold.

**Multivariate logistic regression analysis was applied to adjust the confounders [age, gender, risk habits (cigarette smoking, betel quid chewing and alcohol drinking)] and clinico-pathologic parameters [tumour sizes, lymph node metastasis and pathological tumour staging]

### Pathway and network analysis

The top significant signaling pathway revealed by IPA analysis was identified as integrin-linked kinase signaling pathway (Table 5). Five different groups of molecular and cellular functions were identified and included cell death and survival, cellular function and maintenance, cellular development, cellular growth and proliferation, and cellular movement. Table 6 shows the top three molecular and cellular functions that were associated with amplified and deleted genes. They were cell death and survival, cellular function and maintenance that were mostly associated with colony survival of cells (p = 8.72E-05) through contributions of ATR, CA9, CCND1, FANCG, RAD21, RB1CC1, TERC and TNFSF10 genes. IPA analysis also revealed that there were 26 genes associated with head and neck SCC. These 26 genes were MALAT1, MRC1, POLQ, CCND1, SOX17, LIFR, FGF4, mir-15, FGF3, SHANK2, RAD21, EGFR, FGF19, PPFIA1, TPCN2, MECOM, ANO1, ORAOV1, FADD, DDX58, EPPK1, LYN, ATR, SETD2, MYEOV and CTTN. Network analysis on 1427 genes linked to CNA identified correlation in the most remarkable network with cell death and survival, cellular movement and cellular development (Table 7). This significant network harbored 73 genes and between them, the major centers (cores) like CCND1, RELA, TP63 and EGFR formed interconnected auto-regulatory and feed forward circuitry in the network (S1 Fig). The main function of this network involved tumour growth and proliferation by evasion of apoptosis signals thereby promoting cell survival and metastasis.

**Table 5:** Top significant pathways associated with CNAs associated genes.

Ingenuity Canonical Pathways	-log(p-value)	Molecules
ILK Signaling	3.37E00	RELA,SNAI2,CFL1,ACTB,PPP2R5B,ACTN3,VEGFB,VIM,PIK3R4,RICTOR,PPP1R14B,CCND1,PPP2R3A,RHOD,RHOA,PPM1L,RPS6KA4,PIK3CB,GSK3B,TESK1,ITGB5,MYL3
mTOR Signaling	3.34E00	EIF3H,PPP2R5B,RAC1,VEGFB,EIF3E,PIK3R4,RICTOR,PLD1,FAU,PRKCI,RPS20,EIF3B,PPP2R3A,RHOD,RHOA,PPM1L,PRKAA1,MRAS,RPS6KB2,PIK3CB,RPS6KA4,RPS3
Tight Junction Signaling	2.68E00	RELA,CLDN11,ACTB,HSF1,PPP2R5B,CLDN18,MARK2,CPSF1,RAC1,PRKAR2A,MYLK,GPAA1,PRKCI,PPP2R3A,CLDN1,RHOA,PPM1L,PRKAR1B,MYL3
UVA-Induced MAPK Signaling	2.54E00	TIPARP,PARP15,PARP10,RPS6KB2,MRAS,PLCB3,PIK3CB,RPS6KA4,PIK3R4,PARP9,EGFR,PARP14
Role of CHK Proteins in Cell Cycle Checkpoint Control	2.53E00	PPP2R3A,RAD9A,PPP2R5B,PPM1L,E2F5,ATR,NBN,RAD1,CDC25A

**Table 6:** Top significant molecular and cellular functions associated with CNAs involving associated genes.

Molecular and Cellular Functions	Function annotation	p values	Molecule Genes
Cell Death and Survival	colony survival of cells	1.65E-04	ATR, CA9, CCND1, FANCG, RAD21, RB1CC1, TERC, TNFSF10
	colony survival of tumor cell lines	8.18E-04	CA9, CCND1, FANCG, RAD21, RB1CC1, TERC, TNFSF10
	cell viability of fibroblast cell lines	9.69E-04	ATR, CEBPD, FANCG, MUS81, NBN, RAD9A
	cell survival of cervical cancer cell lines	3.04E-03	KAT5, RAD21
	colony survival of breast cancer cell lines	3.04E-03	CA9, RB1CC1
Cellular Function and Maintenance	colony survival of cells	1.65E-04	ATR, CA9, CCND1, FANCG, RAD21, RB1CC1, TERC, TNFSF10
	colony survival of tumor cell lines	8.18E-04	CA9, CCND1, FANCG, RAD21, RB1CC1, TERC, TNFSF10
	autophagy of epithelial cells	3.04E-03	FADD, TNFSF10
	colony survival of breast cancer cell lines	3.04E-03	CA9, RB1CC1
	uptake of bacteria	4.96E-03	OTUB1, RAC1, RHOA
Drug Metabolism	activation of cytarabine	1.33E-03	ATR, CDC42BPG, NEK11, RPS6KB2, RYK
	synthesis of hydrocortisone	1.54E-03	CYP11B1, CYP11B2, RHOA
	cleavage of hyaluronic acid	8.80E-03	HYAL1, HYAL2
	binding of progesterone	3.94E-02	DNAJA1, STIP1

**Table 7:** Top significant networks and the associated network functions linked with CNAs associated genes.

ID	Top Diseases and Functions	Score	Focus Molecules	Molecules in Network
1	Cell Death and Survival, Cellular Movement, Cellular Development	72	86	14-3-3, 26s Proteasome, ACAD9, ACTB, ADRBK1, ANGPT1, ARRB1, ATR, Actin, Akt, Ap1, BAD, BAG1, BCR(complex), CAMP, CARD11, CARD6, CASR, CCND1, CD3, CD86, CDC25A, CEBPD, CORO1B, CPNE4, CPT1A, CRBN, CTTN, CYP11B2, Caspase 3/7, Cdk, Cofilin, Creb, Cyclin A, Cyclin E, DAB2, DDX58, EGFR, EPPK1, ERK, ERK1/2, F Actin, FADD, FOSL1, FSCN1, Focal adhesion kinase, GDNF, GPER1, GSK3B, Gsk3, HSF1, Hdac, Histone h3, Histone h4, Hsp27, Hsp70, Hsp90, IFN Beta, IKK (complex), IL7R, IgG, Interferon alpha, Jnk, KAT5, LY6K, LYN, MAP2K1/2, MAP3K11, MAP4K2, MBD4, MME, MST1R, MTORC1, MUS81, MYLK, Mek, Mmp, NEU3, NFkB (complex), OVOL1, P2RY2, P2RY6, P38 MAPK, PARP, PDGF BB, PELI3, PI3K (complex), PI3K(family), PIK3CB, PLD1, PLSCR1, PRKAR2A, PRKCI, PRKDC, PRLR, PTGER4, PTP4A3, Pkc(s), Pld, RAC1, RASSF1, RB1CC1, RELA, RHOA, RICTOR, RIPK2, RNA polymerase II, RNF216, RPS3, RUSC2, Rac, Ras, Ras homolog, Rock, SCRIB, SDCBP, SEMA3B, SHARPIN, SKIL, SKP2, SMARCC1, SNAI2, Shc, Smad2/3, TCR, TNFSF10, TP63, TRAIP, TRPC1, UBA7, Ubiquitin, VCP, VEGFB, VIM, VOPP1, Vegf, caspase, estrogen receptor, mir-506, p85 (pik3r)
2	DNA Replication, Recombination, and Repair, Cancer, Cellular Development	41	63	AIFM1, ALG5, ARHGAP21, ARHGEF26, ATAD2, ATAD3B, Alpha tubulin, BARD1, BCL6, BLM, BRMS1, C3orf58, C3orf62, C5orf22, CD72, CEP63, CHCHD6, CNIH2, COX17, CPSF1, CREB5, CSDE1, CTDSPL, CXCL12, CXCR4, CYP27B1, DCSTAMP, DDIT3, DDX11, DDX54, DEPTOR, DGCR8, DISC1, DLEU1, DLEU2, DNAJB5, DRAP1, DROSHA, DTX3L, E2F1, E2F8, E2f, ECT2, EDEM1, EIF3H, EPB41L4AAS1, EXOSC8, FAM162A, FCHSD2, GINS1, GNA12, GNE, GPAA1, GPSM2, GSR, HIST1H1B, HIST1H2AB, HIST1H2AG, HIST1H2BJ, HIST1H3B, HSPH1, KANK2, KIAA0196, KIF20A, KIF22, KLF15, MAFK, MARK2, MCM10, MCM2, MCM4, MCM5, MFAP1, MGLL, MLH1, MMS22L, MTHFD1, MXD1, MYC, MYO9A, NAA40, NCKIPSD, NDE1, NDEL1, NDUFB6, NUPR1, PARP10, PARP14, PARP9, PFKFB4, PITPNM1, POLA2, POLQ, PPFIA1, RAB11FIP5, RACGAP1, RAI14, RARRES1, RASAL2, RBM14, RCL1, RHOA, RMI2, RNF139, RNF169, RPL21, RPS16, RPS27, RRM1, RSL1D1, SAMD4A, SHOX2, SIGMAR1, SKA2, SKP2, SLC25A20, SNRPC, SPIDR, SRGAP2, STAM, TBXA2R, TESK1, TFDP2, TGM2, TMEM126A, TONSL, TRIB1, TRMT13, TSC22D2, TUBGCP5, UMPS, USP36, USP8, VHL, WDR76, XRCC2, XRN1, YWHAG, mir-15, mir-191
3	Cellular Movement, Cell Death and Survival, Cellular Assembly and Organization	40	62	ABCC4, ACPP, ACSL3, AIMP1, AIMP2, AMOTL2, APLP2, AQP3, AR, ARHGEF17, ATP1A1, ATP1B3, ATRIP, AUP1, Actin, B4GALT1, BHLHE40, BUB1, CASP3, CAST, CDC42EP2, CDC42EP4, CDCA5, CDH1, CEL, CENPE, CLCA2, CLRN1, CNBP, COL18A1, CSPG4, DAG1, DARS, DHCR24, DNAJC13, DSE, Dynein, EEF1D, EEF1G, ELK3, EPB41, ERBB2, ESPL1, FANCG, FASN, FAU, FEN1, FKBP4, FNDC3B, FOXA1, FOXH1, FSTL1, GLIPR2, GNAI2, GNB2, GPI, HGF, HLTF, HUS1, HYAL1, ITGB5, KDELR2, KDM4B, KIF22, KPNA1, LIG1, LMBRD2, LRIG1, LTBP3, MAD2L2, MMP16, MSX2, Mre11, NBN, NKX3-1, NPR3, PDGFA, PDIA5, PFN1, PGK1, PKD1, PLCD1, PLEC, PLXDC2, PMEPA1, PODXL, PPID, PRSS3, PSENEN, PTPN23, PTPRF, RAD1, RAD17, RAD9A, RAD9B, RAP2B, RHOD, RNA polymerase I, RNF7, ROR1, RORA, RPA, RPL8, RPN1, RPS16, RPS20, RPS3, RPS3A, Rac, Rnr, SCAP, SEC61A1, SEMA3F, SFRP4, SLC12A6, SLC16A1, SLC3A2, SNX1, SNX2, SNX32, SSH2, SSH3, STT3B, SYVN1, Secretase gamma, TARS, TF, TMEM74, TNFRSF12A, TOPBP1, TOPORS, TOX, TPD52, URI1, UXT, VARS, VIM, YWHAB, ZNF148, miR-1285-3p (and other miRNAs w/seed CUGGGCA)

## Discussion

It has been previously noted that the TCGA consortium [19] had conducted the largest genome-wide profiling study on 172 OSCC samples including oral tongue, buccal mucosa, alveolar ridge, lip, floor of mouth and hard palate using array CGH technology. In order to validate the CNAs derived from this OSCC cohort, we selected samples from within the oral cavity and excluded oropharynx, hypopharynx, and larynx. We identified 26 amplifications and 3 deletions with a frequency of ≥ 8%. Amplifications outnumbered deletions and were noted in chromosomes 3q, 5p, 7p, 8q, 9p, 10p and 11q while deletions were observed in chromosomes 3p and 8p. This study confirms and adds to the earlier evidence of frequent CNAs among OSCCs that have been reported in the TGCA and the International Cancer Genome Consortium (ICGC) [19,20]. As depicted in Fig 2, evidence of replicating recurrent CNAs in the present research was in agreement with those from ICGC and TGCA projects that could provide new insights into oral cancer biology.

To the best of our knowledge this is the first study that have identified the presence of one or more of a group of CNAs (gain 7p, 8q, 11q, and 9p) which function as the novel CNA signature from array CGH analysis. Interestingly, this CNA signature could serve as a clue to determine which OSCC patients have a high risk for lymph node metastasis and therefore an advanced tumor stage. Apart from that, the Kaplan Meier survival curve analysis revealed that presence of one or more of a group of this CNA signature was significantly associated with poor prognosis (p < 0.050). Additionally, Multivariate Cox regression model analysis revealed that this CNA signature group remained as an independent prognostic marker (HRR = 3.455, 95% C1 1.125–10.615, p = 0.026) after adjustment for selected sociodemographic (age, gender, and risk habits) and clinico-pathological parameters (tumor subsite, tumor differentiation, tumor size, lymph node status and tumor staging). The qPCR method was used to further validate the CNAs identified from array CGH. Copy number analysis on LRP12, TPM2, EGFR, FSCN1, CCND1, CLPTM1L, CHL1 and CSMD1 genes were carried out using qPCR. The rationale of choosing these CNA associated genes was basically due to LRP12, TPM2, EGFR, CCND1 being matched with ICGC and TCGA databases whereas FSCN1, CLPTM1L, CHL1 and CSMD1 had been found to be associated with oral cancer [12, 31–33]. This study attempted to elucidate whether these genes were the driver genes in the CNA regions which is 7p, 5p, 3p and 8p. Thus, the validation of these CNA-associated genes resulted in the identification of a “genetic signature” marker. This genetic signature marker contains LRP12, CCND1, EGFR and TPM2 genes that could predict clinical outcomes and facilitate selection of therapeutic strategies in oral cancer management that are tailor-made for patients.

CNAs have the tendency to disrupt proto-oncogenes or tumour suppressor genes, and are known to be major contributors to poor prognosis of oral cancer [7,9,10]. In this current study, we have identified highly frequent copy number alterations in chromosomes 3p, 3q, 8p, 8q and 11q, as described in previous studies that are capable of leading to poor clinical outcome in OSCC [10, 13, 23][10,25,26]. Amplification of 8q22.3-q23.1 was the most frequent event in the current study, and was seen in 18.70% (n = 75) of all OSCCs. Genomic alterations in chromosome 8q, especially amplification of 8q22.3-q23.1, have been commonly noted in OSCC [27]. In this current study, amplification of chromosome 8q was found to be significantly related to advanced pathologic stages in OSCC patients. The plausible reason behind this observation could be attributed by the presence of the putative oncogene known as LRP12 gene which belongs to the LDLR superfamily. According to Garnis et al. [34] suggest that this gene plays a role in oral tumourigenesis and over-expression of this gene is associated with oral cancer.

Losses in 3p26.3-3p26.1 and 3p21.31 were frequently detected and remained as a prognosticator in OSCC cases. These regions harbour the tumour-suppressor gene known as Cell Adhesion Molecule L1 (CHL1), which has been recently shown to contribute in oral tumourigenesis [10]. Loss of this gene able to arrest both in vitro and in vivo proliferation and invasion of tumour cells in breast tumourigenesis [28][29].

Both arms of chromosomes 3 and 8 contain several tumour related genes that are involved in the genesis and progression of oral cancer [8]. Of these, loss in 8p23.32 has been reported more frequently [8]. In this study, deletion of chromosome 8p was found to be associated with advanced pathologic stage of the tumour. The focal area of 8p23.32 that contained the CUB and sushi multiple domain protein 1 (CSMD1) genes could be of interest in future investigations. Deletion and expression loss of this gene have been reported in association with poor survival, lymph node metastasis and advanced pathologic staging in several cancers [30].

Amplification in 11q13.3 was associated with advanced stage of the tumour in OSCC. It has been well-established that chromosome 11q has cancer-related genes that play an important role in tumourigenesis [31,32]. Cancer-related genes including ANO1, CCND1, CTTN, FADD and ORAOV1 are involved in tumour cell proliferation, evasion of apoptosis, invasion, and migration [33–37]. The amplification of 11q13 has been related with poor clinical outcome [38] and metastasis in head and neck cancer [39]. This data reflects the great value of this region as being a valuable biomarker in the prognosis and treatment planning of oral cancers.

Amplification in 7p12-22 has been identified in almost 30% of OSCCs and more than 40% of HNSCC samples from TCGA [25, 35]. We found that amplification in 7p is associated with tumour size (T3-T4) and advanced pathologic staging among OSCCs. Among the candidate oncogenes harbored in this region such as EGFR, TWIST1, and HOXA genes, EGFR has shown a high level of amplification in the OSCC samples of the TCGA project [25]. In this study, amplification in 9p21.1–13.3 was identified in 14.7% of OSCC samples.

Amplification of 9p, especially 9p13, has been frequently reported in OSCC, contributing to early stages of oral tumourigenesis [10, 36]. This current study also revealed that amplification of 9p was significantly associated with lymph node metastasis that might be driven by proto-oncogenes such as CA9, VCP, DCTN3, and STOML2. Towle et al. [36] demonstrated that the inhibition of these putative genes in OSCC cell lines suppressed tumour cell proliferation, suggesting that amplification of 9p13 is more likely to contribute in the aggressiveness of multiple oncogenes in oral tumourigenesis.

Various studies have employed pathway and network analyses to filter the driver genes in signaling pathways and cancer-related gene networks from the robust cancer data sets [37–39]. In this study, several oncogenic signaling pathways were identified using the IPA pathway analysis. The most significant signaling pathway was associated with Integrin-linked kinase (ILK) signaling. Integrin-linked kinase, a candidate oncogene, acts as a multifunctional serine/threonine kinase. Activation of this gene results in cell proliferation, evasion of apoptotic signals and metastasis [40]. Recently, Que et al. [40] showed that targeting the ILK signalling pathway would suppress tumour cell proliferation, the adhesion and invasion ability in oral tumourigenesis and inhibit tumour growth, invasion, and metastasis in the in vivo model. These findings highlight that the ILK signalling pathway plays a novel role in oral tumourigenesis by regulating EMT associated genes and other downstream targets in this pathway. The IPA analysis also showed that the biological functions related to the CNAs associated genes included cell death and survival, cellular function and maintenance, cellular development, cellular growth and proliferation, and cellular movement. All these hallmark cancer-associated functions are well-documented by Douglas Hanahan and Robert Weinberg in their review paper [41]. The identification of the CCND1, an amplified gene harboured in 11q13.3, was involved in all of the findings and implies that its over-expression would lead to the characteristics of cancers by promoting the proliferation, migration, and invasion of tumour cells and the evasion of apoptosis signals in oral tumourigenesis.

The IPA network analysis revealed that a a novel network was related to cell death and survival, cellular movement and cellular development. Within this network, several genes were identified namely CCND1, RELA, TP63, and EGFR as being major contributors to tumour cell proliferation, immortalization, and metastasis in oral tumourigenesis. The interaction network between these four candidate oncogenes namely CCND1, RELA, TP63, and EGFR have been associated with several oncogenic pathways which are the PI3 Kinase/Akt signalling, NF-κB signalling, cell cycle control signalling, and MAPK/Erk ingrowth and differentiation signalling pathways. Overall, these oncogenic pathways cross-regulate each other and are regulated by EGFR, ERK, and Akt phosphorylation, forming an important network that enhances tumor cell activities such as evasion of apoptosis, immortalization, proliferation and metastasis in tumorigenesis.

In summary, this study has recognized several CNAs that are associated with oral tumorigenesis. This study also demonstrated the significant association between amplification of chromosome 8q, 11q, 7p and 9p and deletion of 8p with clinico-pathologic parameters such as the size of the tumour, metastatic lymph nodes and pathological stage in OSCC. Furthermore, co-amplification of the four chromosomes 7p, 8q, 9p, 11q that function as a CNA signature and the genetic markers (CCND1, EGFR, LRP12 and TPM2) remained as independent prognosticators in OSCC for predicting disease outcome. Apart from that, this research detected several copy number changes that are related with pivotal biological networks which often disrupt oral tumorigenesis in different oncogenes associated with CNAs. This research will provide an enhanced appreciation of the CNAs in OSCC and will add to the growing body of knowledge that these pathways/networks play in oral tumorigenesis.

## Supporting information

S1 Table Detailed list of CNAs identified from the current study that shared similarities with the TGCA and ICGC of the oral cancer array CGH OSCC study. (XLSX)

S2 Table Three-year survival rates for the amplification and non-amplification of the selected CNAs. (DOCX)

S3 Table Table of multivariate cox regression model analysis of chromosome 7p, 8q, 9p, 11q and genetic signature in OSCC overall survival. (DOCX)

S1 Fig IPA network analysis. There are total 86 CNA associated genes in top significant network which relate cell death and survival, cellular movement, cellular development. EGFR, RELA, CCND1, TP63 are the main four gene hubs in the network that highlighted in red circle. Among the 86 genes, these genes are known for performing the biological functions by interconnecting and auto-regulating with the four gene hubs including cell proliferation (CCND1, EGFR, MEK, ERK 1/2, AP-1, p85, p38 MAPK, GSK3), metastasis which relate to migration or invasion (RELA, VIM, VCP, FSCN1, SNAI2, FAK, RHOA, RAC, RAC1, ROCK), apoptosis (TP63, RELA, caspase 3/7, BAD, PI3K, ATR, 14-3-3) and angiogenesis (VEGF).
(TIF)
