# Supplementary material for: Genome wide profiling in oral squamous cell carcinoma identifies a four genetic marker signature of prognostic significance
Source: PLoS One. 2017 Apr 6;12(4):e0174865. doi: 10.1371/journal.pone.0174865 (PMC5383235; doi:10.1371/journal.pone.0174865)
Supplement: S2 Table — (DOCX) [file pone.0174865.s002.docx]

S2 Table Three-year survival rates for the amplification and non-amplification of the selected CNAs

| **Analysis model** | 8q | 11q | 9p | 7p | One or more than one marker of chr7p8q9p11q |
| --- | --- | --- | --- | --- | --- |
| **Amplification frequency** | 34.48% | 25.0% | 45.45% | 32.35% | 36.76% |
| **Non-amplification frequency** | 66.13% | 61.86% | 55.52% | 72.93% | 78.85% |
| **Kaplan-Meier analysis** | 0.0043 | 0.0002 | 0.0290 | 0.0003 | 0.0003 |
| **Multivariate Cox regression analysis*** | HRR = 1.677, 95% C1 0.807-3.485, p = 0.166 | (HRR = 3.211, 95% C1 1.417-7.275, p = 0.005) | (HRR = 1.378, 95% C1 0.528-3.596, p = 0.512) | (HRR = 2.022, 95% C1 0.904-4.524, p = 0.087) | (HRR = 3.554, 95% C1 1.161-10.886, p = 0.026) |

* The p-value was adjusted for clinic-pathological and socio-demographical parameters
