# Supplementary material for: Genome wide profiling in oral squamous cell carcinoma identifies a four genetic marker signature of prognostic significance
Source: PLoS One. 2017 Apr 6;12(4):e0174865. doi: 10.1371/journal.pone.0174865 (PMC5383235; doi:10.1371/journal.pone.0174865)
Supplement: S3 Table — (DOCX) [file pone.0174865.s003.docx]

**S3 Table. Multivariate cox regression model analysis of chromosome 7p, 8q, 9p, 11q and genetic signature in OSCC overall survival**

| **Variables** | **Category** | **No. of patients (%)** |  | | | **Variables** | **Category** | **No. of patients (%)** |  | | | **Variables** | **Category** | **No. of patients (%)** |  | | | **Variables** | **Category** | **No. of patients (%)** |  | | | **Variables** | **Category** | **No. of patients (%)** |  | | | **Variables** | **Category** | **No. of patients (%)** |  | | |
| --- | --- | --- | --- | --- | --- | --- | --- | --- | --- | --- | --- | --- | --- | --- | --- | --- | --- | --- | --- | --- | --- | --- | --- | --- | --- | --- | --- | --- | --- | --- | --- | --- | --- | --- | --- |
|  |  |  | **Multivariate Logistic regression**** | | |  |  |  | **Multivariate Logistic regression**** | | |  |  |  | **Multivariate Logistic regression**** | | |  |  |  | **Multivariate Logistic regression**** | | |  |  |  | **Multivariate Logistic regression**** | | |  |  |  | **Multivariate Logistic regression**** | | |
|  |  |  | **OR** | **95% CI** | **p value** |  |  |  | **OR** | **95% CI** | **p value** |  |  |  | **OR** | **95% CI** | **p value** |  |  |  | **OR** | **95% CI** | **p value** |  |  |  | **OR** | **95% CI** | **p value** |  |  |  | **OR** | **95% CI** | **p value** |
| **Total** |  | 68 |  |  |  | **Total** |  | 68 |  |  |  | **Total** |  | 68 |  |  |  | **Total** |  | 68 |  |  |  | **Total** |  | 68 |  |  |  | **Total** |  | 68 |  |  |  |
|  |  |  |  |  |  |  |  |  |  |  |  |  |  |  |  |  |  |  |  |  |  |  |  |  |  |  |  |  |  |  |  |  |  |  |  |
| **chromosome 8q** | **no change** | 46 (61.3) | 1.00† |  |  | **chromosome 7p** | **no change** | 40 (53.3) | 1.00† |  |  | **chromosome 11q** | **no change** | 59 (78.7) | 1.00† |  |  | **chromosome 9p** | **no change** | 64 (85.3) | 1.00† |  |  | **chromosome 8p** | **no change** | 53 (70.7) | 1.00† |  |  | **Genetic signature** | **no marker** | 24 (32.0) | 1.00† |  |  |
|  | **Amplification** | 29 (38.7) | 1.677 | 0.807-3.485 | 0.807 |  | **Amplification** | 35 (46.7) | 2.022 | 0.904-4.524 | 0.087 |  | **Amplification** | 16 (21.3) | 3.211 | 1.417-7.275 | **0.005** |  | **Amplification** | 11 (14.7) | 1.378 | 0.528-3.596 | 0.512 |  | **Deletion** | 22 (29.3) | 3.088 | 1.426-6.689 | **0.004** |  | **≥ 1 marker** | 51 (68.0) | 3.554 | 1.161-10.886 | **0.026** |
|  |  |  |  |  |  |  |  |  |  |  |  |  |  |  |  |  |  |  |  |  |  |  |  |  |  |  |  |  |  |  |  |  |  |  |  |
| **Gender** | **Male** | 26 (34.7) | 1.00† |  |  | **Gender** | **Male** | 26 (34.7) | 1.00† |  |  | **Gender** | **Male** | 26 (34.7) | 1.00† |  |  | **Gender** | **Male** | 26 (34.7) | 1.00† |  |  | **Gender** | **Male** | 26 (34.7) | 1.00† |  |  | **Gender** | **Male** | 26 (34.7) | 1.00† |  |  |
|  | **Female** | 49 (65.3) | 1.053 | 0.436-2.538 | 0.909 |  | **Female** | 49 (65.3) | 0.874 | 0.368-2.077 | 0.761 |  | **Female** | 49 (65.3) | 1.522 | 0.574-4.035 | 0.398 |  | **Female** | 49 (65.3) | 1.063 | 0.431-2.620 | 0.895 |  | **Female** | 49 (65.3) | 1.535 | 0.589-4.001 | 0.38 |  | **Female** | 49 (65.3) | 1.017 | 0.446-2.316 | 0.969 |
|  |  |  |  |  |  |  |  |  |  |  |  |  |  |  |  |  |  |  |  |  |  |  |  |  |  |  |  |  |  |  |  |  |  |  |  |
| **Age (years)** | **<45** | 12 (16.0) | 1.00† |  |  | **Age (years)** | **<45** | 12 (16.0) | 1.00† |  |  | **Age (years)** | **<45** | 12 (16.0) | 1.00† |  |  | **Age (years)** | **<45** | 12 (16.0) | 1.00† |  |  | **Age (years)** | **<45** | 12 (16.0) | 1.00† |  |  | **Age (years)** | **<45** | 12 (16.0) | 1.00† |  |  |
|  | **≥45** | 63 (84.0) | 1.473 | 0.512-4.237 | 0.472 |  | **≥45** | 63 (84.0) | 1.745 | 0.586-5.197 | 0.317 |  | **≥45** | 63 (84.0) | 1.471 | 0.528-4.102 | 0.461 |  | **≥45** | 63 (84.0) | 1.371 | 0.465-4.042 | 0.568 |  | **≥45** | 63 (84.0) | 1.666 | 0.573-4.839 | 0.348 |  | **≥45** | 63 (84.0) | 1.525 | 0.532-4.370 | 0.432 |
|  |  |  |  |  |  |  |  |  |  |  |  |  |  |  |  |  |  |  |  |  |  |  |  |  |  |  |  |  |  |  |  |  |  |  |  |
| **Smoking** | **No** | 52 (69.3) | 1.00† |  |  | **Smoking** | **No** | 52 (69.3) | 1.00† |  |  | **Smoking** | **No** | 52 (69.3) | 1.00† |  |  | **Smoking** | **No** | 52 (69.3) | 1.00† |  |  | **Smoking** | **No** | 52 (69.3) | 1.00† |  |  | **Smoking** | **No** | 52 (69.3) | 1.00† |  |  |
|  | **Yes** | 23 (30.7) | 0.721 | 0.272-1.912 | 0.511 |  | **Yes** | 23 (30.7) | 0.667 | 0.257-1.732 | 0.405 |  | **Yes** | 23 (30.7) | 0.78 | 0.289-2.103 | 0.623 |  | **Yes** | 23 (30.7) | 0.793 | 0.283-2.223 | 0.659 |  | **Yes** | 23 (30.7) | 0.596 | 0.224-1.580 | 0.298 |  | **Yes** | 23 (30.7) | 0.719 | 0.281-1.838 | 0.49 |
|  |  |  |  |  |  |  |  |  |  |  |  |  |  |  |  |  |  |  |  |  |  |  |  |  |  |  |  |  |  |  |  |  |  |  |  |
| **Drinking** | **No** | 64 (85.3) | 1.00† |  |  | **Drinking** | **No** | 64 (85.3) | 1.00† |  |  | **Drinking** | **No** | 64 (85.3) | 1.00† |  |  | **Drinking** | **No** | 64 (85.3) | 1.00† |  |  | **Drinking** | **No** | 64 (85.3) | 1.00† |  |  | **Drinking** | **No** | 64 (85.3) | 1.00† |  |  |
|  | **Yes** | 11 (14.7) | 1.148 | 0.325-4.052 | 0.83 |  | **Yes** | 11 (14.7) | 0.981 | 0.280-3.439 | 0.976 |  | **Yes** | 11 (14.7) | 0.886 | 0.241-3.257 | 0.855 |  | **Yes** | 11 (14.7) | 1.061 | 0.302-3.725 | 0.926 |  | **Yes** | 11 (14.7) | 1.215 | 0.333-4.430 | 0.768 |  | **Yes** | 11 (14.7) | 1.314 | 0.377-4.583 | 0.668 |
|  |  |  |  |  |  |  |  |  |  |  |  |  |  |  |  |  |  |  |  |  |  |  |  |  |  |  |  |  |  |  |  |  |  |  |  |
| **Betel quid chewing** | **No** | 40 (53.3) | 1.00† |  |  | **Betel quid chewing** | **No** | 40 (53.3) | 1.00† |  |  | **Betel quid chewing** | **No** | 40 (53.3) | 1.00† |  |  | **Betel quid chewing** | **No** | 40 (53.3) | 1.00† |  |  | **Betel quid chewing** | **No** | 40 (53.3) | 1.00† |  |  | **Betel quid chewing** | **No** | 40 (53.3) | 1.00† |  |  |
|  | **Yes** | 35 (46.7) | 1.83 | 0.809-4.141 | 0.147 |  | **Yes** | 35 (46.7) | 1.788 | 0.773-4.136 | 0.174 |  | **Yes** | 35 (46.7) | 1.449 | 0.606-3.466 | 0.405 |  | **Yes** | 35 (46.7) | 1.829 | 0.787-4.249 | 0.161 |  | **Yes** | 35 (46.7) | 1.68 | 0.728-3.875 | 0.224 |  | **Yes** | 35 (46.7) | 2.078 | 0.931-4.640 | 0.074 |
|  |  |  |  |  |  |  |  |  |  |  |  |  |  |  |  |  |  |  |  |  |  |  |  |  |  |  |  |  |  |  |  |  |  |  |  |
| **Tumour size** | **T1-T2** | 45 (60.0) | 1.00† |  |  | **Tumour size** | **T1-T2** | 45 (60.0) | 1.00† |  |  | **Tumour size** | **T1-T2** | 45 (60.0) | 1.00† |  |  | **Tumour size** | **T1-T2** | 45 (60.0) | 1.00† |  |  | **Tumour size** | **T1-T2** | 45 (60.0) | 1.00† |  |  | **Tumour size** | **T1-T2** | 45 (60.0) | 1.00† |  |  |
|  | **T3-T4** | 30 (40.0) | 2.767 | 1.013-6.941 | **0.03** |  | **T3-T4** | 30 (40.0) | 2.379 | 0.921-6.144 | 0.073 |  | **T3-T4** | 30 (40.0) | 2.504 | 1.019-6.151 | **0.045** |  | **T3-T4** | 30 (40.0) | 2.963 | 1.158-7.578 | **0.023** |  | **T3-T4** | 30 (40.0) | 3.04 | 1.217-7.594 | **0.017** |  | **T3-T4** | 30 (40.0) | 2.385 | 0.960-5.924 | 0.061 |
|  |  |  |  |  |  |  |  |  |  |  |  |  |  |  |  |  |  |  |  |  |  |  |  |  |  |  |  |  |  |  |  |  |  |  |  |
| **Lymph node metastasis** | **Negative** | 38 (50.7) | 1.00† |  |  | **Lymph node metastasis** | **Negative** | 38 (50.7) | 1.00† |  |  | **Lymph node metastasis** | **Negative** | 38 (50.7) | 1.00† |  |  | **Lymph node metastasis** | **Negative** | 38 (50.7) | 1.00† |  |  | **Lymph node metastasis** | **Negative** | 38 (50.7) | 1.00† |  |  | **Lymph node metastasis** | **Negative** | 38 (50.7) | 1.00† |  |  |
|  | **Positive** | 37 (49.3) | 1.864 | 0.719-4.836 | 0.2 |  | **Positive** | 37 (49.3) | 1.709 | 0.661-4.419 | 0.269 |  | **Positive** | 37 (49.3) | 1.618 | 0.635-4.123 | 0.313 |  | **Positive** | 37 (49.3) | 1.568 | 0.582-4.225 | 0.374 |  | **Positive** | 37 (49.3) | 1.906 | 0.739-4.913 | 0.182 |  | **Positive** | 37 (49.3) | 1.615 | 0.630-4.141 | 0.319 |
|  |  |  |  |  |  |  |  |  |  |  |  |  |  |  |  |  |  |  |  |  |  |  |  |  |  |  |  |  |  |  |  |  |  |  |  |
| **pTNM Staging** | **Early** | 26 (34.7) | 1.00† |  |  | **pTNM Staging** | **Early** | 26 (34.7) | 1.00† |  |  | **pTNM Staging** | **Early** | 26 (34.7) | 1.00† |  |  | **pTNM Staging** | **Early** | 26 (34.7) | 1.00† |  |  | **pTNM Staging** | **Early** | 26 (34.7) | 1.00† |  |  | **pTNM Staging** | **Early** | 26 (34.7) | 1.00† |  | 0.214 |
|  | **Advanced** | 49 (65.3) | 2.467 | 0.524-11.616 | 0.253 |  | **Advanced** | 49 (65.3) | 2.774 | 0.610-12.620 | 0.187 |  | **Advanced** | 49 (65.3) | 3.121 | 0.676-14.413 | 0.145 |  | **Advanced** | 49 (65.3) | 3.058 | 0.657-14.220 | 0.154 |  | **Advanced** | 49 (65.3) | 2.67 | 0.577-12.355 | 0.209 |  | **Advanced** | 49 (65.3) | 2.652 | 0.569-12.363 |  |

CI: confidence interval

† Reference category

Significant p - value were highlighted in bold.

**Multivariate logistic regression analysis was applied to adjust the confounders [age, gender, risk habits (cigarette smoking, betel quid chewing and alcohol drinking)] and clinico-pathologic parameters [tumour sizes, lymph node metastasis and pathological tumour staging]
