# Supplementary figures and images for: Genome wide profiling in oral squamous cell carcinoma identifies a four genetic marker signature of prognostic significance
Source: PLoS One. 2017 Apr 6;12(4):e0174865. doi: 10.1371/journal.pone.0174865 (PMC5383235; doi:10.1371/journal.pone.0174865)

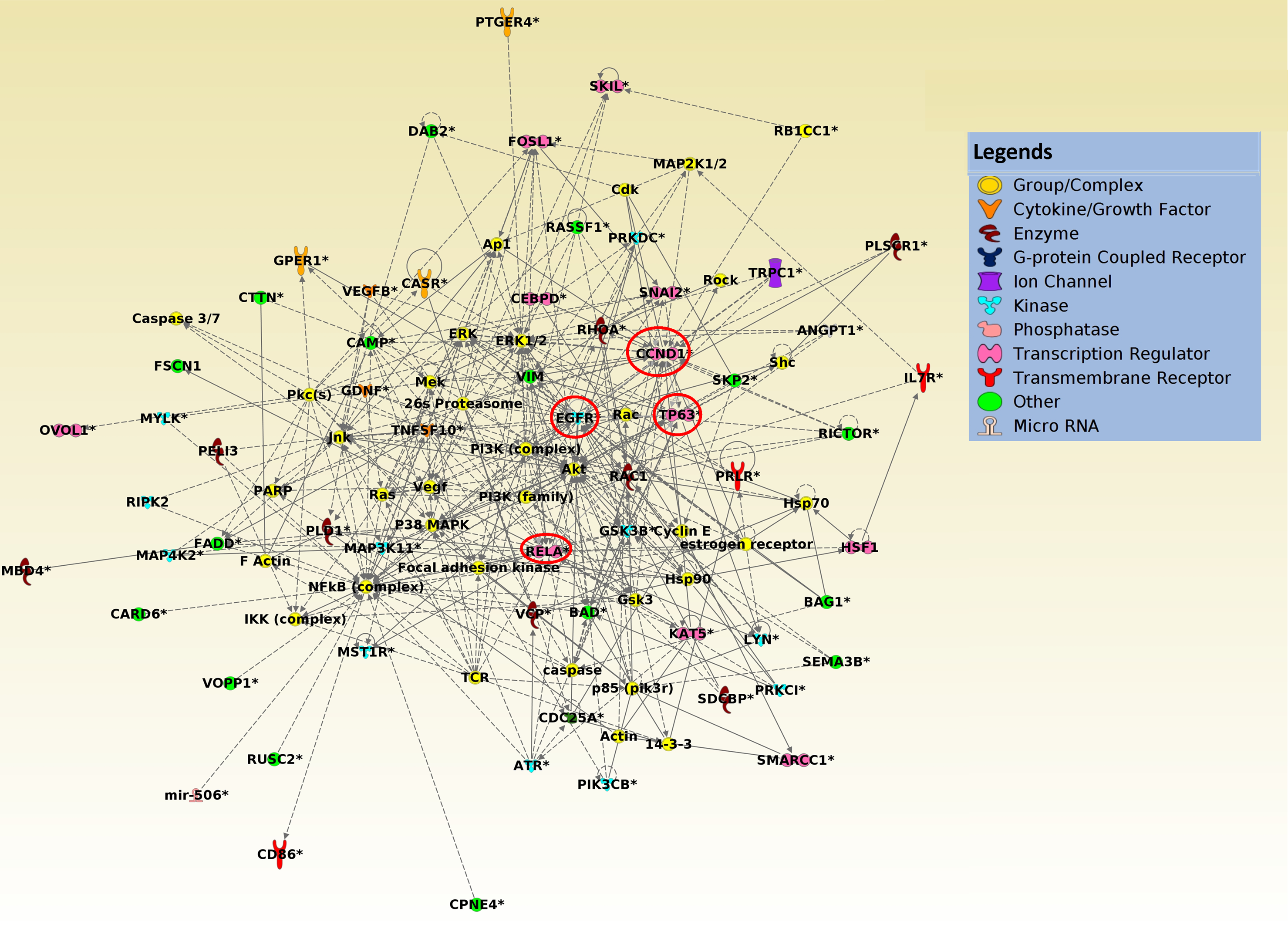

Supplement: S1 Fig — There are total 86 CNA associated genes in top significant network which relate cell death and survival, cellular movement, cellular development. EGFR, RELA, CCND1, TP63 are the main four gene hubs in the network that highlighted in red circle. Among the 86 genes, these genes are known for performing the biological functions by interconnecting and auto-regulating with the four gene hubs including cell proliferation (CCND1, EGFR, MEK, ERK 1/2, AP-1, p85, p38 MAPK, GSK3), metastasis which relate to migration or invasion (RELA, VIM, VCP, FSCN1, SNAI2, FAK, RHOA, RAC, RAC1, ROCK), apoptosis (TP63, RELA, caspase 3/7, BAD, PI3K, ATR, 14-3-3) and angiogenesis (VEGF). (TIF) [file pone.0174865.s004.tif]
